# Anti‐AHNAK1 Antibodies Are a Novel Diagnostic Biomarker for Systemic Lupus Erythematosus

**DOI:** 10.1155/bmri/6381475

**Published:** 2025-08-28

**Authors:** Yasushi Matushita, Kazuhisa Nozawa, Kentaro Doe, Yoshinari Takasaki, Ken Yamaji, Naoto Tamura

**Affiliations:** ^1^ Department of Rheumatology, School of Medicine, Juntendo University, Bunkyo, Tokyo, Japan, juntendo.ac.jp; ^2^ Department of Internal Medicine, Juntendo University Koshigaya Hospital, Koshigaya City, Saitama, Japan

**Keywords:** AHNAK1, autoantibodies, autoantigens, systemic lupus erythematosus

## Abstract

Calcium signaling is essential for the proper function of immune cells. Recent studies have shown that the scaffold protein, AHNAK1, is important for efficient calcium signaling and NFAT activation in T cells through its ability to properly localize calcium ion (Ca^2+^) channels at the plasma membrane. Interestingly, both T cells and B cells of systemic lupus erythematosus exhibit activation signaling anomalies with dysregulated Ca^2+^ response, and enhanced Ca^2+^ signaling has emerged as a target for treatment with SLE. Therefore, we hypothesized SLE patients may have autoantibodies (Abs) against AHNAK1 because anti‐AHNAK1 antibodies possibly are able to interfere with Ca^2+^ signaling through binding to AHNAK1, subsequently resulting in aberrant T cells signal transduction. In the present study, we notably found that sera from SLE patients profoundly elicit immunoreaction against AHNAK1 when compared to normal healthy controls (NHCs) or patients with other systemic autoimmune diseases, such as polymyositis/dermatomyositis (PM/DM), systemic sclerosis (SSc), Sjögren’s syndrome (SjS), mixed connective tissue disease (MCTD), and rheumatoid arthritis (RA). Additionally, the expression level of AHNAK1 in peripheral blood mononuclear cells (PBMCs) from SLE patients was significantly increased compared to NHCs. We propose that measurement of serum anti‐AHNAK1 antibodies can be used as a possible biomarker for the diagnosis of SLE. In addition, our data suggest that AHNAK1 antibodies may have an indicative role in the pathogenesis of SLE.

## 1. Introduction

Systemic lupus erythematosus (SLE) is a systemic autoimmune disease with a distinct pathology and multiple organ involvement. A great number of antibodies against multiple intracellular autoantigens are frequently found in their sera as unique characteristics of SLE. Among the serum circulating Abs, several Abs with disease specificity are found in SLE, such as the double‐stranded DNA [1]. Although these Abs have been indicated to play important roles for SLE development, the specific pathogenic roles for SLE still are not fully elucidated [2]. Therefore, various studies of these Abs have influenced understanding of the disease pathogenesis [3].

In addition, there are a number of factors that contribute to the pathogenesis of SLE other than Abs. Among these factors, a great number of studies have indicated the importance of dysfunctions of lymphocytes for SLE pathogenesis [4–6]. For instance, many studies have shown abnormal cytokine production and aberrant cell signaling in T cells of SLE patients [7]. In lymphocyte activation, phosphorylation of several kinases localized at downstream sites of TCR is mediated through TCR stimulation. For example, the reduction of the expression level of LCK, which is a member of the Src kinase family, has been reported in T cells from SLE patients [8]. On the other hand, the increased expression of Syk kinase has been reported in B cells from SLE patients [9] resulting in the discovery of novel potential therapeutics for SLE, fostamatinib, and Syk kinase inhibitor [10, 11]. These tyrosine kinases promote calcium ions (Ca^2+^) influx in T cells from extracellular to intracellular space through the calcium released activated channel (CRAC) on the cell membrane. Interestingly, a number of recent studies have shown aberrant Ca^2+^ signaling occurs in lymphocytes in SLE, resulting in disorder in their lymphocytes [12]. Therefore, aberrant Ca^2+^ signaling has implied one of the important factors for SLE pathogenesis. Interestingly, increased expression of NFATc2, which belongs to the nuclear factor of activated T cells (NFAT) family, was reported in the nuclei of activated T cells from SLE patients after CD3 stimulation [13]. Moreover, increased expression of NFATc1 has also been reported in lupus‐prone MRL/lpr mice [14]. Therefore, calcineurin inhibitors (cyclosporine and tacrolimus), which have been developed for the treatment of other autoimmune diseases, are considered to be effective in the treatment of SLE [15].

AHNAK1 belongs to AHNAK, consisting of two giant proteins (700 kDa), AHNAK1/desmoyokin and AHNAK2. AHNAK1 has been reported to be localized in both the cytoplasm [16] and the cell membrane. AHNAK1 is a scaffold protein and a critical component for Ca^2+^ signal transduction through stabilization of CRAC on the plasma membrane [17]. AHNAK1 has been shown to be highly expressed in naïve CD4^+^ T cells, but not CD8^+^ T cells [17], and mature cytotoxic lymphocytes (CTLs) can express AHNAK1 especially strongly after stimulation [18]. Moreover, in AHNAK1^−/−^ mice, CD4^+^ T cells poorly respond to TCR stimulation with low proliferation and low IL‐2 production [19] and CD8^+^ CTLs show marked reduction in granzyme‐B production, cytolytic activity, and IFN‐*γ* secretion after TCR stimulation [18]. Therefore, we hypothesized that SLE patients may have Abs against AHNAK1 because anti‐AHNAK1 antibodies possibly are able to interfere with Ca^2+^ signaling through binding to AHNAK1, subsequently resulting in aberrant T cell signal transduction.

## 2. Materials and Methods

### 2.1. Patients and Sera

All study participants received care at Juntendo University Hospital. The sera with connective tissue diseases were collected and stored at −80°C until use. The ethics committee of Juntendo University Hospital approved this study (Approval Number: 17‐251). Sera from patients with SLE (*n* = 61), systemic sclerosis (SSc; *n* = 40), polymyositis/dermatomyositis (PM/DM; *n* = 40), Sjögren’s syndrome (SjS; *n* = 40), mixed connective tissue disease (MCTD; *n* = 40), and rheumatoid arthritis (RA; *n* = 40) were evaluated in addition to normal healthy controls (NHCs; *n* = 118). All patients were diagnosed with the necessary criteria [20–25].

### 2.2. Enzyme‐Linked Immunosorbent Assay (ELISA)

An ELISA protocol described previously [26, 27] was used with slight modifications. Briefly, diluted AHNAK1 recombinant protein (Abcam, Cambridge, MA, United States) in phosphate‐buffered saline (PBS) (100 ng/mL) was coated in Immulon 2 microtiter plates (Dynatech Laboratories, Alexandria, VA, United States) (100 *μ*L/well) overnight at 4°C. Diluted human sera in PBS (1:200) was then added to the wells. Diluted horseradish peroxidase (HRP)–conjugated goat anti‐human IgG (Caltag Laboratories, Carlsbad, CA, United States) (1:5000) was used to detect the bound antibodies in the sera, and then 2,2 ^′^‐azino‐bis(3‐ethylbenzthiazoline‐6‐sulfonic acid) (ATBS) was added for the detection. Samples in duplicate were monitored on optical densities (ODs) at 405 nm.

### 2.3. Indirect Immunofluorescence (IIF) Microscopy

HEp‐2 cell substrate (MBL, Nagoya, Japan) was stained by diluted mouse anti‐AHNAK1 monoclonal antibodies (1:200) (Abcam) or diluted anti‐AHNAK1 Abs from positive patients sera (1:200) in ELISA. The bound antibodies were detected with Alexa‐488‐conjugated anti‐human IgG antibodies and Alexa‐594‐conjugated anti‐mouse IgG antibodies (Invitrogen, Grand Island, NY, United States) at a 1:500 dilution for 1 h, respectively. The images were captured and analyzed by a fluorescence microscope (Keyence Biorevo, BZ‐9000, Osaka, Japan) at 200× magnification.

### 2.4. Quantitative Real‐Time Reverse Transcription Polymerase Chain Reaction (RT‐PCR)

PBMCs were purified from blood samples of SLE patients and NHCs. Total RNA was isolated using the RNeasy Mini Kit (Qiagen). cDNA was synthesized using a PrimeScript RT Reagent kit (Takara Bio Inc., Shiga, Japan), and quantitative real‐time RT‐PCR was then performed using SYBR Premix Ex Taq (Takara Bio Inc.) according to the manufacturer’s instructions. The primers for human *β*‐actin (purchased from Takara Bio Inc.) and human AHNAK1 (purchased from Origene Technology Inc., Rockville, MD, United States) (Product Number: HP205444) were used for RT‐PCR. The amplification cycles consisted of 95°C for 10 s as the first step (one cycle); 95°C for 5 s and 65°C for 30 s as the second step (40 cycles); and finally 95°C for 15 s, 60°C for 60 s, and 95°C for 15 s as the third step (one cycle). The sample was normalized by the expression of human *β*‐actin transcripts for the determination of the quantitative expression levels of the transcripts.

### 2.5. Statistical Analysis

Experimental data were statistically compared with the Mann–Whitney *U*‐test for differences in quantitative parameters and Fisher’s exact test for differences in qualitative parameters, and *p* < 0.05 was considered statistically significant.

## 3. Results

### 3.1. Increased AHNAK1 Immunoreactivity in SLE

**Figure 1:**
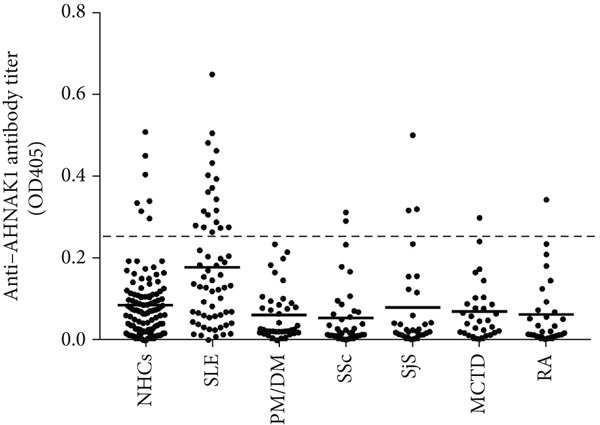
AHANK1 immunoreactivity in connective tissue diseases. AHNAK1 immunoreactivity in sera with systemic lupus erythematosus (SLE; *n* = 61), polymyositis/dermatomyositis (PM/DM; *n* = 40), systemic sclerosis (SSc; *n* = 40), Sjögren’s syndrome (SjS; *n* = 30), mixed connective tissue disease (MCTD; *n* = 30), rheumatoid arthritis (RA; *n* = 30), and healthy controls (*n* = 115) was evaluated by ELISA. Dots indicate the serum antibody titer (OD_405_) for each patient of the groups. The cutoff value (dotted line) for positive reaction was designed as value above the mean OD of normal healthy controls plus two standard deviations (SDs) of NHCs (0.268 at OD_405_).

To determine whether AHNAK1 was a possible target of autoimmunity in SLE, we evaluated the titer of anti‐AHNAK1 antibodies in sera obtained from patients with SLE or other connective tissue diseases, as well as NHCs (Figure 1 and supporting information (available here)). As there was no designated standard for cutoff values for positive AHNAK1 antibody reactivity, we provisionally defined the cutoff value for a positive reaction as the mean OD of NHCs plus two standard deviations (SDs) in the same manner as our previous reports [26, 27]. According to the definition, 0.268 OD at 405 nm (mean OD for NHCs, 0.086; SD for NHCs, 0.091) was used as the cutoff value for a positive reaction in the present study. Notably, anti‐AHNAK1 antibody levels in ODs were significantly higher in patients with SLE when compared to healthy controls and the other control groups (*p* < 0.01). In addition to the level of anti‐AHNAK1 antibodies, the positivity rate for serum anti‐AHNAK1 antibodies was significantly greater in SLE compared to NHCs and the other control groups as well (Table 1) when the cutoff value for a positive reaction was designed as the mean OD of NHCs plus two SDs. We also investigated the correlation between anti‐AHNAK1 antibody levels and the disease activity in chronologically collected sera before and after the treatment; however, no significant correlation was recognized (data not shown).

**Table 1:** Prevalence of serum anti‐AHNAK1 antibodies in connective tissue diseases.

**Connective tissue diseases**	**SLE**	**PM/DM**	**SSc**	**SjS**	**MCTD**	**RA**	**NHCs**
Patient number	61	40	40	30	30	30	115
Anti‐AHNAK1 Abs ‐positive patients (%)	17 (27.9%)	0 (0%)	2 (5%)	3 (10%)	1 (0%)	1 (3.3%)	7 (3.5%)
Statistical analysis (vs. SLE)	ND	*p* < 0.01	*p* < 0.01	*p* < 0.01	*p* < 0.01	*p* < 0.01	*p* < 0.01
Statistical analysis (vs. NHCs)	*p* < 0.01	*p* = 0.17	*p* = 0.12	*p* = 0.07	*p* = 0.13	*p* = 0.11	ND

*Note:* Experimental data were statistically compared by Fisher’s extract test, and differences with *p* values < 0.05 were considered to be statistically significant. The cutoff value for positive reaction was designed as value above the mean OD of normal healthy controls plus two standard deviations (SDs) of NHCs.

Abbreviations: MCTD, mixed connective tissue disease; NHCs, normal healthy controls; PM/DM, polymyositis/dermatomyositis; RA, rheumatoid arthritis; SjS, Sjögren’s syndrome; SLE, systemic lupus erythematosus; SSc, systemic sclerosis.

### 3.2. Characteristics of SLE Patients With AHNAK1 Antibodies

The clinical findings in SLE patients with or without anti‐AHNAK1 antibodies (*n* = 17 or 42, respectively) are summarized in Table 2. We found the mean age at the time of both sampling and onset was younger in anti‐AHNAK1 antibody positive SLE patients compared to anti‐AHNAK1 antibody negative SLE patients, although the disease duration was not statistically different. In terms of SLE manifestations, the frequency of lymphopenia is lower in anti‐AHNAK1 antibody positive SLE patients (13/17, 76.4%) compared to anti‐AHNAK1 antibody negative SLE patients (40/42, 95.2%), although no significant differences were observed in other disease manifestations. We also investigated the correlation between anti‐AHNAK1 antibody levels and other components of Table 2, and we could not find any correlation.

**Table 2:** Patient’s profile of SLE patient with or without anti‐AHNAK1 antibodies.

	**Anti-AHNAK1 antibodies (+)**	**Anti-AHNAK1 antibodies (−)**
Numbers of patients	17	42
Gender		
Female	14	37
Male	3	5
Age at time of sampling (mean, IQR)	37.6 (27.8–43.5)	52.5 (44.0–63.0) ^∗∗^
Age at onset (mean, IQR)	21.1 (16.8–25.2)	32.1 (22.5–38.0) ^∗∗^
Disease duration (mean, IQR)	16.5 (10.75–18.0)	20.3 (15.0–27.0)
SLE manifestations	12	28
Rash	13	27
Nephritis	13	40 ^∗^
Lymphopenia	1	2
Hemolytic anemia	5	16
Thrombocytopenia	9	31
Arthritis	4	4
Serositis	2	5
CNS involvement	12	28

*Note:* Patient’s clinical profile at the same time of their serum sample collection was analyzed for comparison between anti‐AHNAK1 positive and negative SLE patients. A number of the patients with SLE manifestations were shown. The continuous variables (age and disease duration) were statistically analyzed by the Mann–Whitney test. The frequency of each SLE manifestation was statistically analyzed by Fisher’s exact test.

Abbreviation: IQR, interquartile range.

∗ indicates statistical difference (*p* < 0.05). ∗∗ indicates statistical difference (*p* < 0.01).

### 3.3. The Staining Pattern of Anti‐AHNAK1 Antibodies in IIF Microscopy

We investigated a staining with anti‐AHNAK1 antibody positive sera detected by ELISA using IIF microscopy on human HEp‐2 cell substrate (Figure 2b). We confirmed that serum anti‐AHNAK1 antibodies react with cytoplasmic molecules, and a similar staining pattern was observed in commercial anti‐AHNAK1 monoclonal antibodies (Figure 2a) suggesting that anti‐AHNAK1 antibodies belong to cytoplasmic auto Abs.

Figure 2Staining with anti‐AHANK1 antibodies in IIF analysis. (a) Staining with anti‐AHNAK1 antibodies was evaluated by indirect immunofluorescence microscopy. (b) Staining with anti‐AHNAK1 positive prototype serum showed cytoplasmic staining in HEp‐2 substrate similar to that with anti‐AHNAK1 monoclonal antibodies.

**Figure:**
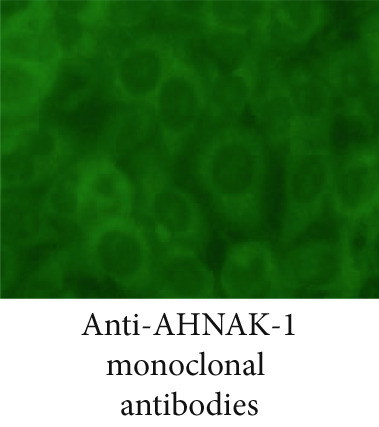
(a)

**Figure:**
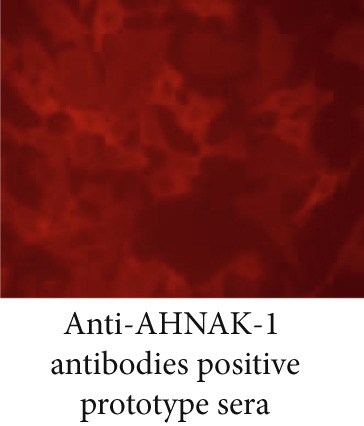
(b)

### 3.4. Increased AHNAK1 Expression in Peripheral Blood Mononuclear Cells of SLE Patients

**Figure 3:**
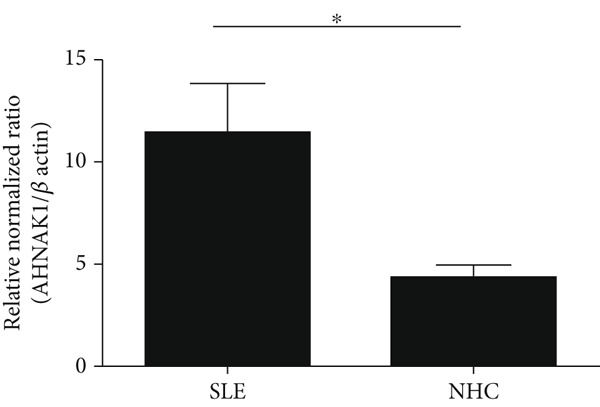
Increased expression of AHNAK1 in PBMCs of SLE patients. Gene expression of AHNAK1 was evaluated by quantitative RT‐PCR in PBMCs of the patients with SLE and NHCs. The expression of AHNAK1 was significantly increased in SLE compared with NHCs. Statistical analysis was performed using the Mann–Whitney *U*‐test against SLE. Bars in indicate SD. *p* values < 0.05 ( ^∗^) was considered as statistically significant.

To determine whether AHNAK1 relates to SLE pathogenesis, we evaluated AHNAK1 expression in PBMCs derived from SLE patients. As expected, increased AHNAK1 expression in PBMCs was observed in SLE patients compared to NHCs (Figure 3).

## 4. Discussion

The study demonstrated the presence of SLE‐specific anti‐AHNAK1 antibodies compared to other CTDs. In addition, the staining with anti‐AHNAK1 antibodies showed an intracellular cytoplasmic staining pattern.

It is not clear why SLE patients frequently elicit an autoimmune reaction towards AHNAK1 compared to NHCs in addition to other connective tissue diseases. In the present study, we found that AHNAK1 expression in PBMCs was rich compared to those of NHCs. As abnormal increased apoptosis of lymphocytes was considered to be one of the pathogenesis of SLE [28], intracellular AHNAK1 may be excessively released from apoptotic cells into extracellular space, resulting in an autoimmune reaction towards AHNAK1. Furthermore, native protein structure has been reported to possibly undergo posttranslational modification during apoptosis. The modified antigens might stimulate the immune system, subsequently resulting in autoantibody production if the immune system undergoes a proinflammatory situation such as SLE. The other possible explanation is that AHNAK1 may constitute a protein complex with other proteins that are recognized as autoantigens in SLE. For example, proliferating cell nuclear antigen (PCNA) constitutes a protein complex with other proteins, and it is known to be one of the autoantigens frequently recognized in patients with SLE. We have previously demonstrated that patients with SLE frequently have autoantibodies against not only PCNA but also multiple other PCNA binding proteins of the PCNA‐protein complex in vivo, suggesting that epitope spreading predominantly occurs in SLE. AHNAK1 might be involved in the protein complex with other proteins recognized as autoantigens in SLE, such as PCNA. Therefore, anti‐AHNAK1 antibodies may be useful as specific biomarkers for the diagnosis of SLE.

Regarding the possibility of a pathological role for SLE pathogenesis, we found that AHNAK1 was highly expressed in PBMC of SLE patients compared to NHCs. Downregulation of expression of T cell receptor *ζ* chain (TCR *ζ* chain) expression, which is involved in T cell activation signaling pathway, has been reported as pathogenesis for SLE. In addition, hypermethylation, which represses gene expression, has also been reported within CD3*ζ* gene promoter in SLE patients. Furthermore, increased recruitment of NFATc2, which is a member of the NFAT family, is observed in the nuclei of activated T cells of SLE patients after CD3 stimulation. These reports suggest that abnormal signal transduction in T cells plays an important role in the pathogenesis of SLE. AHNAK1 has been reported to be one of the important molecules in the stabilization of the structure of the Ca^2+^ channel. Interestingly, Li et al. reported that CRAC promoted the progression of lupus nephritis as administration of YM‐58483, an inhibitor of the CRAC, reduced the concentration of antidouble strand DNA antibodies and immune deposition in the glomeruli. Furthermore, ion channels, such as the transient potential receptor canonical‐channel 6 (TRAP6) which is important for the control of calcium currents, have been reported as possible influencers of the secretory profile of SLE lymphocytes. Therefore, the increased expression of AHNAK1 in PBMCs of SLE patients may affect the regulation of Ca^2+^ signaling, consequently resulting in the development of SLE. Furthermore, we found the mean age at the time of disease onset was younger in anti‐AHNAK1 antibody positive SLE patients compared to anti‐AHNAK1 antibody negative SLE patients. In terms of SLE manifestations, the frequency of lymphopenia is lower in anti‐AHNAK1 antibody positive SLE patients compared to anti‐AHNAK1 antibody negative SLE patients. Although the interpretation of these data is difficult, autoantibodies against AHNAK1 may affect the physiological function of AHNAK1 in lymphocytes, subsequently developing the disease progression of SLE.

There are a few limitations to this study. First, this study involves small sample size data, and it is a retrospective analysis. The small sample size might limit the diagnostic applicability of serum anti‐AHNAK1 antibodies. Due to the retrospective nature of our study, there might be limitations in ascertaining variables that might influence the results. Second, we hypothesized that anti‐AHNAK1 antibodies might affect the Ca^2+^ balance of lymphocytes in SLE patients, resulting in the pathogenesis of SLE, as we found that the mRNA level of AHNAK1 was highly expressed in lymphocytes of SLE patients; however, we could not perform the functional assay of anti‐AHNAK1 antibodies using lymphocytes of SLE patients. Third, although we showed high immunoreactivity of SLE patients against AHNAK1 recombinant protein in ELISA, we could not confirm these results in immunoblot or immunoprecipitation analysis for the detection of the native form protein of AHNAK1. The reason why we could not successfully perform immunoblot or immunoprecipitation analysis may be explained by the fact that AHNAK1 is a high molecular weight protein (approximately 700 kDa). This kind of molecule might not be suitable for immunoblot or immunoprecipitation analysis because of its huge molecular weight. However, we confirmed that staining of serum anti‐AHNAK1 antibodies in human HEp‐2 cells substrate and the staining pattern (cytoplasmic pattern) were identical for commercial anti‐AHNAK1 monoclonal antibodies. The result indicates that serum anti‐AHNAK1 antibodies react not only to the recombinant protein but also to the native form of AHNAK1.

The theme of this study is AHNAK1, but at the present time, there is no previous study focusing on the AHNAK1 molecule in SLE patients; the content of the study is original and unique. The whole picture of its pathological condition of SLE has not been elucidated, and if this antibody is associated with a specific clinical symptom, it is important because it may contribute greatly to the elucidation of its etiology and its treatment.

In conclusion, anti‐AHNAK1 antibodies may be useful as one of the diagnostic biomarkers for SLE as the antibodies are rarely recognized in other CTDs. Moreover, AHNAK1 may have a pathogenetic role for SLE through interfering with Ca^2+^ signals of lymphocytes.

**Nomenclature**
SLE: systemic lupus erythematosus
MCTD: mixed connective tissue disease
NPSLE: neuropsychiatric SLE
PCNA: proliferating cell antigen
PM/DM: polymyositis/dermatomyositis
RA: rheumatoid arthritis
SjS: Sjögren’s syndrome
SSc: systemic sclerosis
CRAC: calcium released activated channel

## Disclosure

This study has been presented as a conference in “International Journal of Rheumatic Diseases 2017; 20 (Suppl. 1): 17–131” according to the following link: https://onlinelibrary.wiley.com/doi/10.1111/1756-185X.13178.

## Conflicts of Interest

The authors declare no conflicts of interest.

## Funding

No funding was received for this manuscript.

## Supporting information


**Supporting Information** Additional supporting information can be found online in the Supporting Information section. Titer of serum anti‐AHNAK1 antibodies in connective tissue diseases.

## Data Availability

The all data (Figures 1, 2, and 3, Tables 1 and 2, and Supplemental Description) used the support the findings of this study are within the article and available from the corresponding author upon request without any restriction. The all data used the support the findings of this study are original based on this research project.
