# Supplementary material for: Anti‐AHNAK1 Antibodies Are a Novel Diagnostic Biomarker for Systemic Lupus Erythematosus
Source: Biomed Res Int. 2025 Aug 28;2025:6381475. doi: 10.1155/bmri/6381475 (PMC12407309; doi:10.1155/bmri/6381475)
Supplement: Supplementary file 1 — Supporting Information Additional supporting information can be found online in the Supporting Information section. Titer of serum anti‐AHNAK1 antibodies in connective tissue diseases. [file BMRI-2025-6381475-s001.docx]

Supplymentary Information file

The all data (Figure 1-3, Table 1-2, and Supplemental Description) used the support the findings of this study are within the article and available from the corresponding author upon request without any restriction. The all data used the support the findings of this study are original based on this research project.

Corresponding author:

Kazuhisa Nozawa MD. PhD.

Department of Internal Medicine, Juntendo University Koshigaya Hospital, 660 Fukuroyama, Koshigaya city, Saitama, Japan, 343-0032,

E-mail: [k-nozawa@juntendo.ac.jp](mailto:k-nozawa@juntendo.ac.jp)

Tel: +81-48-975-0321

Fax: +81-3-5800-4893

Supplemental Description. Titer of serum anti-AHNAK1 antibodies in connective tissue diseases

| Connective Tissue Diseases | **SLE** | **PM/DM** | **SSc** | **SjS** | **MCTD** | **RA** | **NHCs** |
| --- | --- | --- | --- | --- | --- | --- | --- |
| Patient number | 61 | 40 | 40 | 30 | 30 | 30 | 115 |
| Optical densities  (mean ± SD) | 0.179  ±  0.149 | 0.006  ±  0.006 | 0.055  ±  0.078 | 0.081  ±  0.119 | 0.071  ±  0.072 | 0.064  ±  0.083 | 0.086  ±  0.092 |
| Statistical analysis  (vs. SLE) | ND | P < 0.01 | P < 0.01 | P < 0.01 | P < 0.01 | P < 0.01 | P < 0.01 |
| Statistical analysis  (vs. NHCs) | P < 0.01 | P = 0.166 | P = 0.384 | P=0.132 | P = 0.435 | P = 0.461 | ND |

SLE, systemic lupus erythematosus; PM/DM, polymyositis/dermatomyositis; SSc, systemic sclerosis; SjS, Sjögren’s syndrome; MCTD, mixed connective tissue disease; RA, rheumatoid arthritis; NHCs, Normal Healthy Controls. Experimental data were statistically compared by Mann–Whitney U test and differences with P-values < 0.05 were considered to be statistically significant.
